# Climate change and its impacts on the world of work

**DOI:** 10.47626/1679-4435-2024-223

**Published:** 2024-11-14

**Authors:** Andrea Franco Amoras Magalhães

**Affiliations:** Editor-in-Chief of the Brazilian Journal of Occupational Medicine

The term “climate change” refers to significant, long-lasting changes in the Earth’s weather patterns. Its origin is directly related to the evolution of environmental science and climatology over time. Climate change has resulted in extreme weather and climate conditions worldwide, as evidenced by the increased frequency and severity of heat waves, heavy precipitation, forest fires, droughts, and tropical cyclones.^1,2^

Climate change has significant impacts on human health, and various academic and institutional references address these issues comprehensively. These sources provide a solid foundation for understanding the diverse impacts of climate change on human health. Some studies estimate that 3.6 billion people already live in areas highly susceptible to climate change. Between 2030 and 2050, climate change is expected to cause approximately 250,000 additional deaths per year from malnutrition, malaria, diarrhea, and heat stress alone. Climate change affects human health by influencing the spread of vectors, water quality, food production, and contributing to air pollution.^3,4^

Climate change can significantly affect the health of 70% of workers worldwide.^1^ Climate fluctuations disrupt our circadian cycle, which regulates the mental and physical states that occur within 24 hours. The financial impacts are also considerable, due to productivity losses, interruptions in economic activities, and damage to infrastructure.^3^

Numerous health effects have been associated with climate fluctuations, including cardiovascular and respiratory diseases, kidney dysfunction, altered mental health, heatstroke, allergies, and mosquito-borne diseases such as dengue and malaria. Extreme weather events can lead to forced displacement of populations, increased exposure to ultraviolet (UV) radiation, air pollution, exposure to pesticides in the workplace, and food shortages that result in hunger and malnutrition.^5^

The working class is increasingly exposed to serious health risks related to climate change, challenging occupational safety and health measures to prioritize worker protection. It will be necessary to review existing legislation or create new regulations and guidelines.^1,2^

Protecting workers from the effects of climate change is crucial, especially as extreme weather events and other changes in the working environment become more frequent. Comprehensive strategies to protect workers’ health and lives must be developed by companies in collaboration with leadership, employees, occupational health staff, and other professionals. These strategies include:

1. Risk assessment and planning
  - Climate risk assessment: conduct assessments to identify specific risks related to climate change in the workplace, such as exposure to high temperatures, extreme events and changes in air quality.
  - Emergency planning: develop and implement emergency and natural disaster response plans that include clear guidelines on how to protect workers during extreme weather events.
2. Protection against extreme heat
  - Labor policies: establish policies that limit exposure to heat, such as adjusting work schedules to avoid the hottest part of the day.
  - Breaks and hydration: provide regular breaks and access to drinking water. Create shaded, cool areas for resting.
  - Personal protective equipment (PPE): Provide adequate protective clothing and, if necessary, body cooling systems.
3. Protection against extreme weather events
  - Training and equipment: provide training on how to respond during extreme events such as storms, floods, and hurricanes. Ensure that workers have access to adequate protective equipment and can shelter in safe places.
  - Infrastructure maintenance: keep infrastructure and work equipment in good condition to minimize damage and ensure safety during extreme weather events.
4. Air quality and pollution
  - Air quality monitoring: install systems to monitor air quality and minimize exposure to pollutants, especially in industries such as construction and mining.
  - Adequate ventilation: ensure good ventilation in workplaces to reduce the concentration of pollutants in the air.
5. Health and well-being
  - Health programs: encourage health programs that include regular assessments, access to medical care, and psychological help workers cope with stress related to extreme weather events.
  - Education and training: offer training on the effects of climate change on health and how to prevent climate-related diseases.
6. Infrastructure security and resilience
  - Infrastructure assessment: assess and strengthen infrastructure to withstand extreme weather events. This may include constructing more resilient buildings and maintaining effective drainage systems.
  - Business recovery plans: develop strategies to ensure business recovery and worker safety in the event of extreme weather events.
7. Policies and regulations
  - Legal compliance: ensure that safety practices comply with local and state regulations on occupational health and safety.
  - Advocacy and collaboration: partner with unions, associations, and other organizations to promote better worker protection policies related to climate change.
8. Technology and innovation
  - Monitoring technologies: develop the use of advanced technologies to monitor environmental conditions and predict extreme weather events.
  - Innovative solutions: encourage the development of innovative solutions to protect workers, such as smart clothing and equipment that helps regulate body temperature.

Implementing these strategies helps to mitigate the adverse risks of climate change, ensuring a safer and healthier working environment. Climate change and its impacts on the world of work also need to be prioritized on the political agenda so that we can discuss new health and safety policies with society and increase the visibility of human health and, particularly, the health of workers, setting better standards of protection.
